# Progress in the role of nanoparticles in the diagnosis and treatment of bone and joint tuberculosis

**DOI:** 10.3389/fmed.2025.1536547

**Published:** 2025-01-24

**Authors:** Yitong Ding, Baiyun Li, Yangfei Yi, Can Liu, Jie Wen, Xiaohong Jian, Yufei Li

**Affiliations:** ^1^Department of Anatomy, Hunan Normal University School of Medicine, Changsha, Hunan, China; ^2^Department of Nursing, Hunan Normal University, Changsha, Hunan, China; ^3^Department of Pediatric Orthopedics, Hunan Provincial People’s Hospital, The First Affiliated Hospital of Hunan Normal University, Changsha, Hunan, China

**Keywords:** bone and joint tuberculosis, nanomaterials, diagnosis, treatment, tuberculosis

## Abstract

Bone and joint tuberculosis (BJTB), caused by Mycobacterium tuberculosis (MTB), is a prevalent form of extrapulmonary tuberculosis that poses significant challenges to global public health due to difficulties in early diagnosis, prolonged treatment cycles, and drug resistance. Recent advancements in nanotechnology have introduced novel solutions for the early detection and precise treatment of BJTB, leveraging unique physicochemical properties such as high specific surface area, targeted delivery capabilities, sustained drug release, and excellent biocompatibility. In diagnostic applications, nanomaterials markedly enhance the sensitivity and accuracy of detection methods while reducing testing time. These technologies are adaptable to resource-limited settings, enabling earlier patient intervention and mitigating disease progression risk. In therapeutic applications, nanomaterials prolong drug retention in bone tissue through targeted delivery, thereby decreasing medication frequency and minimizing toxic side effects, which significantly improves treatment efficacy. Despite substantial progress, further research is required to address long-term safety concerns, broaden clinical applicability, and evaluate performance under complex pathological conditions. This review summarizes recent advancements in nanomaterials for diagnosing and treating BJTB and identifies key areas for future research, laying the groundwork for advancing precision medicine and personalized treatments.

## 1 Introduction

Tuberculosis (TB) is an infectious disease with a rich historical background, dating back to the identification of spinal tuberculosis in Egyptian mummies from 3,000 BC (1). Mycobacterium tuberculosis (Mtb) is the main pathogen of tuberculosis, which spreads through the air and is engulfed by macrophages after entering the lungs. Some pathogens escape from the immune system and spread to the whole body, causing extrapulmonary tuberculosis. Bone and joint tuberculosis are more common, mainly involving the spine and large joints, which can lead to dysfunction and decreased quality of life in severe cases. Despite significant advancements in modern medicine for TB prevention and treatment, the global incidence remains high, with over 10 million new infections reported annually (2). The occurrence of TB is closely associated with socioeconomic development levels, particularly affecting vulnerable populations such as the elderly and individuals living with AIDS who face a significantly higher risk of infection (3–5). Pulmonary tuberculosis remains the most prevalent form, whereas bone and joint tuberculosis ranks third among extrapulmonary cases, underscoring its clinical significance (6). The immunosuppressive effects of HIV substantially exacerbate the spread and progression of tuberculosis (7). Research data indicate that HIV-positive individuals have a 1.42-fold higher risk of developing TB compared to HIV-negative individuals (8). This association underscores the critical role of the immune system in defending against Mycobacterium tuberculosis (Mtb) and highlights the complexity and severity of HIV/TB co-infection on a global scale.

The rising prevalence of multidrug-resistant tuberculosis (MDR-TB) presents significant challenges in the diagnosis and management of bone and joint tuberculosis, often resulting from incomplete or inadequate treatment regimens (9–11). Traditional diagnostic methods are limited by prolonged processing times, while treatment options are constrained by poor drug penetration into bone tissue and the extended duration required for therapy. Additionally, adverse effects associated with antituberculosis drugs can lead to reduced patient adherence to treatment protocols (12). These challenges have spurred researchers to investigate novel therapeutic strategies to enhance clinical outcomes for patients with bone and joint tuberculosis.

In this context, nanomaterials have introduced novel possibilities for the treatment of bone and joint tuberculosis. Nanomaterials possess distinctive characteristics, including enhanced targeted drug delivery, adjustable drug release rates, prolonged drug exposure, and improved drug absorption (13). They can serve as slow-release delivery systems (14, 15) for precise drug administration to infected bone tissue, thereby significantly enhancing drug permeability and therapeutic efficacy while effectively reducing toxicity (16, 95).

Considering the potential of nanomaterials in treating bone and joint tuberculosis and their profound impact on human health, it is crucial to explore their mechanisms of action and potential applications. This article comprehensively examines the pathogenesis, traditional diagnosis and treatment approaches for bone and joint tuberculosis while focusing on the advancements made in utilizing nanomaterials in this field. Furthermore, future research directions and challenges are anticipated.

## 2 Bone and joint tuberculosis

### 2.1 Pathogenesis of bone and joint tuberculosis

**FIGURE 1:**
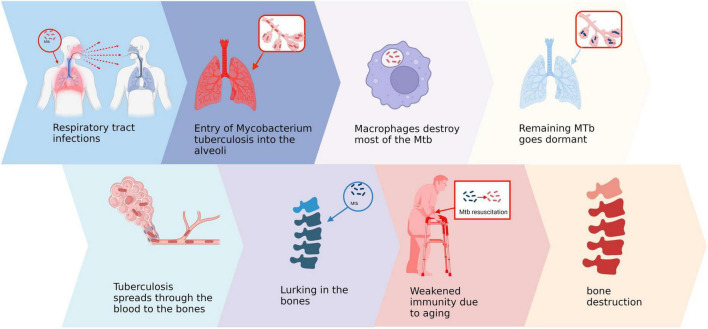
Pathogenesis of bone and joint tuberculosis. Created in BioRender. ding, y. (2025) https://BioRender.com/v66b991.

Bone and joint tuberculosis is a widespread secondary extrapulmonary form of tuberculosis, primarily caused by local inflammation resulting from the hematogenous spread of Mycobacterium tuberculosis (Mtb) to bones or joints (17). The respiratory tract serves as the main route for Mtb transmission, wherein inhalation of droplets containing bacteria leads to colonization in the alveoli (18, 19). Macrophages attempt to eliminate invading Mtb by recruiting immune cells like monocytes, lymphocytes, and neutrophils (20). However, certain bacteria can evade clearance by the immune system, forming granulomas that restrict their proliferation and enter a dormant state (21). The effectiveness of host cell-mediated immune response during this stage plays a crucial role in determining disease control or progression (22). Subsequently, when host immunity declines significantly due to factors such as HIV infection or aging over subsequent years or even decades (23). There are two primary modes of transmission. Firstly, during the initial pulmonary infection, Mtb disseminates via the bloodstream to the bone and enters a dormant state. Under appropriate conditions, this latent infection within the bone tissue can be directly reactivated. Secondly, latent infections in the lungs may reactivate and subsequently spread to the bones through either the bloodstream or the lymphatic system (22). Regardless of the form, Mtb tends to invade the spine and large joints following its entry into bone tissue (24). Upon entering the joint cavity, Mtb typically initiates erosion from surrounding synovial tissues before gradually spreading into cartilage and bone tissues (Figure 1). This erosive process often persists for several months leading to severe disruption of local tissue architecture (25).

Given the aforementioned pathological process, early diagnosis and timely intervention play a pivotal role in managing bone and joint tuberculosis. By promptly identifying and treating the condition, disease progression can be effectively halted, mitigating irreversible tissue damage and ultimately enhancing patient prognosis and quality of life (26). Consequently, it is imperative to explore efficient and accurate early diagnostic technologies as well as optimize treatment plans to address the challenges posed by bone and joint tuberculosis (27).

### 2.2 Diagnosis of bone and joint tuberculosis

The clinical manifestations of bone and joint tuberculosis are intricate and diverse, with spinal tuberculosis being the most prevalent, followed by tuberculous arthritis and extracorporeal tuberculous osteomyelitis (28). Currently, the diagnosis of bone and joint tuberculosis primarily relies on comprehensive approaches such as medical history collection, imaging examination, and hematologic analysis (29). However, these methods have certain limitations at different disease stages, which can impact the accuracy and timeliness of diagnosis. In the early stages of the disease, patients often present with localized dull pain and swelling alone, potentially accompanied by constitutional symptoms like fever, night sweats, and anemia that are associated with tuberculosis (30). Nevertheless, these symptoms lack specificity and can be easily mistaken for other conditions. Furthermore, patients with bone or joint tuberculosis frequently exhibit pathological changes such as caseous necrosis that hinder prompt detection through techniques like smears or bacterial culture—although considered diagnostic gold standards—due to their time-consuming nature often taking weeks or longer (31, 32).

Therefore, the early diagnosis of bone and joint tuberculosis poses not only significant challenges but also a high rate of misdiagnosis. Untimely identification and treatment of bone and joint tuberculosis can result in severe consequences. For instance, when lesions affect the nervous system, patients may experience irreversible neurological damage leading to functional impairment (33, 34). In advanced stages of the disease, patients commonly present with pronounced joint pain, deformity, dislocation, and substantial limitations in daily activities. Imaging techniques such as X-rays often reveal marked reduction in joint space accompanied by destructive changes (35). Progression of the disease may even lead to fibrous ankylosis of the joint resulting in complete loss of joint function (36).

In conclusion, multiple factors limit early-stage diagnosis of bone and joint tuberculosis while advanced stages significantly impact patient quality of life and functional prognosis. Henceforth, optimizing existing diagnostic methods and exploring novel techniques hold great significance for enhancing clinical outcomes.

### 2.3 Treatment of bone and joint tuberculosis

Currently, the management of bone and joint tuberculosis primarily involves first-line anti-tuberculosis drugs, second-line anti-tuberculosis drugs, and surgical intervention when necessary. The foundation of routine therapy consists of first-line agents such as isoniazid (INH), pyrazinamide (PZA), rifampin (RIF), and ethambutol (EMB) (37). Second-line drugs are employed in cases where initial treatment fails or resistance develops. These include injectable agents (streptomycin, kanamycin, amikacin, capreomycin, and viomycin), fluoroquinolones (e.g., ofloxacin, levofloxacin, gatifloxacin, and moxifloxacin), as well as other oral agents (e.g., ethionamide, prothionamide, cycloserine, terizidone, and p-aminosalicylic acid) (38, 39). According to current standards, treatment duration for bone and joint tuberculosis is prolonged. Typically, the minimum duration for antituberculosis drug therapy is 8 months; however, a complete course of chemotherapy requires at least 20 months (40). Nevertheless, this extended treatment period poses a significant challenge to patient adherence. Many patients may discontinue treatment due to stress, resulting in the emergence and dissemination of drug-resistant strains. This has become one of the major challenges in global tuberculosis management (41).

There are various routes of administration for anti-tuberculosis drugs, each with its own set of advantages and disadvantages (42, 43). Oral administration is the most commonly employed method; however, it exhibits slow efficacy and low bioavailability. High oral doses not only contribute to the proliferation of drug-resistant bacteria but also lead to significant toxic side effects such as hepatotoxicity and gastrointestinal discomfort (44). In contrast, parenteral administration offers improved absorption and effectiveness but necessitates the presence of a healthcare professional, often causing discomfort and pain during the procedure. Surgical intervention is recommended in selected cases, particularly for complex situations like deformity correction or neurological impairment, as it can promptly alleviate pain and restore function. Nevertheless, surgical treatment is not universally suitable and typically limited to conditions where clear indications exist, such as established deformities or neurologic impairments (45, 46).

In conclusion, current treatment options partially control bone and joint tuberculosis; however, their limitations are evident. Prolonged treatment duration coupled with drug toxicity leads to poor patient compliance while drug resistance exacerbates treatment challenges. Although surgery may serve as an adjunctive therapy under specific circumstances, it does not apply universally to all patients (Table 1). Therefore, future research should focus on developing more efficient and tolerable treatment strategies along with optimizing existing therapeutic approaches.

**TABLE 1:** General treatment and limitations of bone and joint tuberculosis.

Treatment plan	Concrete method	Advantages	Limitations
Oral administration of anti-tuberculosis drugs	First-line drugs such as isoniazid, rifampicin and ethambutol	Mature and stable	Leads to drug resistance and toxic side effects
Parenteral administration of anti-tuberculosis drugs	First-line drugs such as isoniazid, rifampicin and ethambutol	Better drug absorption and efficacy	Nurses need to be present and administration is painful
Surgery	Selection of internal fixation segment, Debridement of TB lesion, Bone grafting.	Rapidly relieves patients’ pain and significantly improves functioning	Limitations of use
Anti-tuberculosis drug delivery by nanomaterials	BSA NPs, liposome-hydrogel systems, etc.	High potency, less frequent dosing, long drug release, low toxicity	Long-term efficacy and safety are unknown

## 3 Application of nanomaterials in bone and joint tuberculosis

With the rapid advancement of science and technology, nanotechnology has emerged as a frontier in modern medicine with great potential and application value (47, 48). Due to their unique physicochemical properties such as high specific surface area, good biocompatibility, targeting ability, and controlled drug release capability, nanomaterials play a crucial role in the diagnosis and treatment of bone and joint tuberculosis (49, 50). Nanomaterials can be classified into two categories: organic and inorganic (51). Organic nanomaterials like liposomes have gained significant attention due to their excellent biosafety and compatibility (52); however, inorganic nanomaterials such as mesoporous silica are more prominent for bone tissue targeted therapy because of their high stability and drug delivery efficiency (53).

**FIGURE 2:**
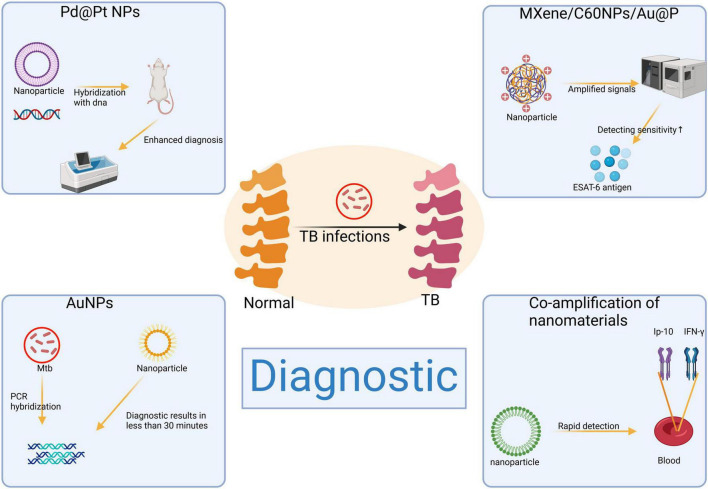
Application of nanomaterials in the diagnosis of bone and joint tuberculosis. Created in BioRender. ding, y. (2025) https://BioRender.com/f06p848.

In the field of diagnostics, several novel nanotechnologies have significantly enhanced the sensitivity and specificity of tests (Figure 2). Within the therapeutic domain, nanomaterials present patients with more efficient and safer treatment alternatives by optimizing targeted delivery systems (54). In recent years, extensive exploration and development of various nanoformulations have led to their clinical application in bone and joint tuberculosis management (55, 56). These nanomaterials not only augment the therapeutic efficacy of antituberculosis drugs within bone tissue but also substantially reduce drug frequency and dosage, thereby mitigating the risk of drug resistance and toxicity (57, 58). Furthermore, integration of nanomaterials into scaffolds provides porous carriers that enhance biocompatibility while promoting bone tissue regeneration (59). These groundbreaking innovations hold immense potential for early detection and effective treatment of bone and joint tuberculosis, ultimately improving patient prognosis and quality of life.

## 4 Diagnostic Nanomaterials in bone and joint tuberculosis

### 4.1 Pd-Pt bimetallic nanoparticles

Palladium-platinum bimetallic nanoparticles (Pd@Pt NPs) have garnered considerable attention as an emerging nanotechnology due to their exceptional performance in catalysis and biosensing applications. The incorporation of palladium into platinum, forming a bimetallic structure, endows Pd@Pt NPs with peroxidase-like properties and significantly enhances the reactive surface area when interacting with substrates such as 3,3′,5,5′-tetramethylbenzidine (TMB) (60). Compared to alternative materials, Pd@Pt NPs exhibit several distinct advantages, such as superior sensitivity, rapid detection capabilities, cost-effectiveness, portability, and visualization features (61). Its porous structure not only offers a high specific surface area but also demonstrates superior catalytic activity and efficiency, thereby significantly enhancing detection performance (62, 63). Furthermore, serving as nanozymes enables them to exhibit long-term storage stability and withstand extreme environments effectively expanding their range of applications (64). Although Pd@Pt NPs have shown great potential in the fields of catalysis and biosensing, the complex preparation process and strict conditions limit their feasibility in large-scale applications.

In Cy et al.’s study (65), Pd@Pt nanoparticles were integrated into a multilayered paper-based assay device and DNA hybridization assay system for the detection of Mycobacterium tuberculosis (Mtb). The results demonstrated that the Pd@Pt nanoparticles exhibited superior catalytic efficiency and enabled target DNA detection within 15 min compared to conventional methods. This diagnostic approach holds significant potential in resource-limited medical settings, providing an efficient and convenient tool for detecting tuberculosis in remote or developing areas. Early diagnosis of bone and joint tuberculosis can reduce bone destruction and improve patient prognosis.

### 4.2 Gold nanoparticles probe

Gold nanoparticles (AuNPs) have emerged as a superior alternative to traditional fluorescence and isotope technology for nucleic acid detection in recent years due to their exceptional stability, efficient nucleic acid hybridization ability, straightforward operational process, making them a rapid and reliable tool (66). Compared to conventional techniques, AuNPs can provide results within approximately 30 min and have been extensively employed for pathogen nucleic acid detection including Mtb (67). However, due to the high cost associated with Au as a precious metal, the widespread adoption of AuNPs in large-scale clinical diagnostics is constrained. In a recent study, Pedrosa et al. (68) simultaneously amplified the rpoB531 and inhA C-15T genes of Mtb using multiplex PCR and utilized gold nanoparticle probe chips for detecting drug resistance gene mutations. The findings demonstrated that this method exhibited a detection consistency rate of up to 100% for rifampin-resistant and sensitive samples while achieving an accuracy rate of 84% for isoniazid-resistant samples, thereby highlighting the potential clinical application value of gold nanoparticle probes in rapidly identifying gene mutations in Mtb.

### 4.3 MXene/C60NPs/Au@P

Enhanced diagnostic methods for bone and joint tuberculosis are urgently needed to overcome the limitations of low sensitivity in traditional approaches. Despite the potential of antigen-detection techniques, there remain significant challenges in isolating and preserving highly specific monoclonal antibodies (69). By integrating nanomaterials into aptamer sensors, these issues can be effectively addressed, leading to a substantial improvement in antigen detection efficiency (70).

In Huang et al.‘s (71) study, researchers successfully developed a dual-signal output aptamer sensor based on MXene/C60NPs/Au@Pt nanocomposites for ultrasensitive detection of ESAT-6 antigen. Leveraging its exceptional REDOX activity and catalytic properties, this nanocomposite exhibited superior specificity and accuracy in distinguishing healthy donors from patients with other lung diseases as well as TB patients. Experimental results demonstrated an impressive system sensitivity of 97.5% and specificity of 96.7%, surpassing that achieved by conventional diagnostic methods. This innovative sensor represents a valuable tool for efficient and reliable clinical diagnosis of bone and joint tuberculosis while also holding great promise for early screening, precision diagnosis, and treatment strategies. However, there are still some potential problems that need to be solved in the practical application of this nanocomposite. For example, its biocompatibility and safety for long-term use have not been fully studied, and there may be a risk of cytotoxicity or environmental effects.

### 4.4 Dual targets for ultrasensitive fluorescence quantification by synergistic amplification of nanomaterials

Enhanced ultrasensitive fluorescence quantification through synergistic amplification of nanomaterials enables dual targeting for improved diagnostic performance in tuberculosis (TB). Traditional diagnostic methods, such as Mtb culture, CT imaging studies, tuberculin skin tests (TST), and interferon-γ release assays (IGRAs), have inherent limitations (72). To overcome these limitations and enhance sensitivity and specificity for TB infection detection, additional biomarkers have been integrated into IGRAs. Notably, the upregulation of IFN-γ-inducible protein 10 (IP-10) in response to Mtb stimulation offers a promising avenue for higher accuracy. The combined detection of IFN-γ and IP-10 further enhances the ability to differentiate active TB cases from healthy individuals (73).

In Shi et al.‘s (74) study, a dual-target strategy was implemented to simultaneously detect IP-10 and IFN-γ in patient blood samples using selective recognition of fluorescent nanomaterials and enzyme-free nucleic acid amplification. Compared to the conventional IGRA method, this approach reduces detection time by at least 12 h and significantly enhances diagnostic efficiency through the specific recognition of aptamers. This methodology presents a novel avenue for rapid diagnosis and clinical decision-making in cases of bone and joint tuberculosis. However, compared to traditional methods, this strategy may necessitate more advanced technical and equipment support, which presents additional challenges for implementation in primary care settings.

## 5 Therapeutic nanomaterials in bone and joint tuberculosis

### 5.1 Chitosan nanoparticles

Chitosan, a naturally-derived biopolymer obtained through N-deacetylation of chitin, can be extracted from crustaceans and aquatic microorganisms (75). Chemically, chitosan is a linear binary heteropolysaccharide composed of β-1,4-linked D-glucosamine (deacetyl unit) and N-acetyl-D-glucosamine (acetyl unit) (76). Due to its exceptional antibacterial properties, chitosan exhibits significant activity against Gram-negative bacteria, Gram-positive bacteria, and fungi (77, 78). Moreover, the drug carrier potential of chitosan is greatly enhanced by its pH-dependent transport capacity, providing it with a notable advantage in pharmaceutical applications (79). The natural glycosaminoglycan structure of chitosan contributes to its excellent biocompatibility as it facilitates easy breakdown and absorption within the human body (80, 81).

The delivery of rifampin was targeted using a chitosan nanoparticle system in a study (82), wherein the conjugation of mannose as a ligand to chitosan significantly enhanced the uptake efficiency of particles by macrophages. By combining this system with an *in situ* gel prepared by Poloxamer 407 and HPMC K4M, the drug release time was extended to 40 h. In a simulated synovial fluid model, the system exhibited preferential uptake by macrophages and efficient release of rifampicin, resulting in Mycobacterium tuberculosis (Mtb) eradication. Compared to conventional drug administration, chitosan nanoparticles demonstrate superior efficacy and reduced toxicity, exemplifying successful integration of materials science and pharmacy for bone and joint tuberculosis treatment. Nevertheless, the application of chitosan still faces several significant challenges. Firstly, given that chitosan is primarily derived from crustaceans, its potential to induce allergic reactions warrants careful consideration. Additionally, its long-term biocompatibility and potential toxicity have not been thoroughly and systematically evaluated. In particular, high-dose or prolonged use may pose hidden risks of cytotoxicity or environmental impact.

### 5.2 Bovine serum albumin nanoparticles

l Bovine serum albumin (BSA) is an acidic protein that is widely distributed in the body fluids and tissues of mammals (83). It consists of a single polypeptide chain with three distinct domains containing multiple drug-binding sites, rendering it an optimal vehicle for drug delivery (84). Due to their ready availability, biocompatibility, lack of immunogenicity, and high drug-binding capacity, BSA nanoparticles (BSA NPs) are extensively employed for drug delivery purposes (85, 86). Traditional oral anti-tuberculosis drugs face challenges in effectively reaching bone tissue due to their rapid degradation rate; however, BSA NPs can specifically target liver and spleen tissues, prolonging the residence time of drugs and significantly enhancing their utilization (87).

In a 12-week controlled trial conducted in a New Zealand white rabbit model of spinal tuberculosis (88), the study demonstrated that BSA NPs significantly outperformed conventional treatments in drug delivery efficiency. Pathological examinations revealed a substantial increase in drug concentration in both bone tissue and blood when delivered via BSA NPs. After 12 weeks of treatment, imaging results indicated complete disappearance of broken bone tissue at the lesion site, with surrounding abscesses and necrotic tissues being fully replaced by normal bone tissue, leading to complete lesion resolution. In contrast, the conventional treatment group exhibited markedly inadequate efficacy. The control group showed incomplete repair of the vertebral body and persistent pathological necrotic nodules in the paravertebral area, underscoring the limited effectiveness of traditional treatment regimens for spinal tuberculosis. These findings highlight the significant advantages of BSA NPs in enhancing drug accumulation at the lesion site and improving tissue repair, thereby offering new insights and potential for more effective treatment of spinal tuberculosis. Nevertheless, certain limitations remain in the clinical application of BSA NPs. For instance, under extreme pH or elevated temperature conditions, their structural integrity may be compromised, thereby impacting the stability of drug delivery and therapeutic efficacy.

### 5.3 Mesoporous silica nanoparticles

Mesoporous silica (MSN) is a versatile material characterized by its porous structure, which exhibits a large pore volume (up to 2.5 cmł/g), tunable pore size ranging from 1.3 to 50 nm, high specific surface area (>1,000 m^2^/g), and exceptional chemical and biological stability (52, 89). These remarkable properties of MSNs enable efficient loading of therapeutic agents, including small molecules, genes, peptides, and proteins, for targeted delivery through electrostatic adsorption or chemical bonding (90, 91).

In a recent study (59), an innovative MSN-based bioscaffold hydrogel system was developed for the controlled release of rifampin and levofloxacin. The results demonstrated that the hydrophobic interaction between the drugs and mesoporous silica nanoparticles (MSNs) significantly extended the drug release duration to approximately 60 days, while effectively mitigating drug resistance and suppressing pathogen proliferation. This strategy exemplifies successful integration of biomaterials with nanotechnology and provides a promising solution for treating bone and joint tuberculosis. However, mesoporous silica nanoparticles (MSNs) exhibit limited biodegradability, and their long-term accumulation in the body may pose toxicity risks, which represent significant challenges that must be addressed prior to clinical application.

### 5.4 Tetracycline modified nanoparticles

Tetracycline (TC) has emerged as an optimal antibacterial agent for targeted bone tissue therapy owing to its strong affinity with hydroxyapatite. It exhibits significant antimicrobial activity against Gram-positive and Gram-negative bacteria, as well as other pathogens, by inhibiting bacterial protein synthesis (92). The distinctive characteristic of tetracycline lies in its selective accumulation in bone tissue, which is further enhanced when combined with nanoparticles, thereby improving the efficiency of targeted drug delivery (93, 94). Polylactic-co-glycolic acid (PLGA) is a hydrophobic synthetic polymer that has gained widespread use in the medical field owing to its superior biodegradability and biocompatibility. The degradation products of PLGA can be safely eliminated via metabolic pathways, rendering it an ideal material for drug delivery. TC-PLGA nanoparticles (TC-PLGA NPs) were synthesized via esterification between the hydroxyl group of tetracycline (TC) and the carboxyl group of PLGA. This system integrates the bone-targeting properties of tetracycline with the advantageous characteristics of PLGA, thereby demonstrating significant potential in the treatment of bone-related diseases (52).

In a study conducted by Liang (95), tetracycline-modified nanoparticles were synthesized using DCC/NHS technology, while rifampicin was employed as the loading drug for control experiments. The experimental results demonstrated that rifapentine-loaded NPs exhibited sustained drug release characteristics for approximately 100 h, in contrast to free rifapentine. The results demonstrated that this nanoparticle system could preferentially accumulate in bone tissue without inducing apparent toxicity and significantly enhance the therapeutic efficacy of the drug. Compared to conventional anti-tuberculosis treatment approaches, this system reduces both the frequency and dosage of drugs administered while minimizing the risk of toxicity, thus providing a more efficient option for treating bone and joint tuberculosis. However, this technique is not without limitations. Firstly, the synthesis process of TC-PLGA nanoparticles is relatively intricate and demands stringent preparation conditions, potentially complicating large-scale production. Secondly, while preliminary experiments have demonstrated low short-term toxicity, further research is necessary to investigate long-term biocompatibility and metabolic characteristics to ensure safety for extended use.

### 5.5 Liposome-hydrogel-drug delivery system

Liposomes are biocompatible delivery systems composed of one or more amphoteric phospholipids, and their unique bilayer structure enables the efficient delivery of both hydrophilic and hydrophobic drugs (96–98). Liposomes possess the ability to encapsulate not only hydrophilic substances within the vesicle core but also hydrophobic materials within a lipid bilayer (99). This exceptional characteristic renders liposomes an outstanding carrier for drug delivery (97) as well as an excellent sustained-release system (100), thereby enhancing drug availability by preventing premature decomposition (101).

In Liu et al.’s (102) studies, a liposome-hydrogel nanoparticle-based sustained-release system was developed and its efficacy was evaluated in New Zealand white rabbits and SD rats. By incorporating liposomes into the hydrogel matrix, this system achieves prolonged and controlled release of isoniazid derivatives while maintaining localized drug concentrations. *In vivo* drug release experiments demonstrated that the drug concentration in the control group rapidly declined to below 0.3 μg/mL within 24 h, whereas the drug concentration in the liposome-hydrogel system was sustained between 0.48 and 0.61 μg/mL, indicating markedly superior stability. The experimental findings demonstrated that this strategy significantly enhances sustained drug release performance and effectively reduces the frequency of drug administration without altering the properties of the hydrogel. Consequently, this approach offers additional therapeutic options for bone and joint tuberculosis treatment, particularly for patients requiring long-term medication management. Although the liposome-hydrogel system demonstrates superior drug delivery performance, the mechanical strength and degradation rate of the hydrogels can vary across different physiological environments. Further optimization is essential to ensure their reliability under diverse pathological conditions. Different nanoparticles used for bone and joint tuberculosis treatment was summarized in Table 2.

**TABLE 2:** Different nanoparticles used for bone and joint tuberculosis treatment.

References	Nanomaterials	Effect
Cy et al. (65)	Pd@Pt NPs	Enhanced diagnostic effectiveness
Pedrosa et al. (68)	AuNPs	Reduced diagnostic time
Huang et al. (71)	MXene/C60NPs/Au@P	Enhanced diagnostic sensitivity and specificity
Shi et al. (74)	Dual targets for ultrasensitive fluorescence quantification by synergistic amplification ofnanomaterials	Reduced diagnostic time
Prabhu et al. (82)	Chitosan nanoparticles	Prolonged drug release
Ma et al. (88)	Bovine serum albumin nanoparticles	Significantly higher concentrations of transported drugs in bone and blood
Yahia et al. (59)	Mesoporous silica nanoparticles	Prolonged drug release
Liang et al. (95)	Tetracycline modified nanoparticles	Enhancing drug efficacy
Liu et al. (69)	Liposome-hydrogel-drug delivery system	Prolonged drug release

## 6 Conclusion and future prospects

Although nanotechnology has demonstrated significant potential in the diagnosis and treatment of bone and joint tuberculosis, its widespread application still encounters numerous challenges (103). The short experimental duration hinders the complete verification of nanomaterials’ efficacy in long-term treatment, as well as the clarification of their potential toxicity and long-term safety. These uncertainties impede the full clinical implementation of this technique. Therefore, further systematic and long-term experiments are necessary to explore biocompatibility, metabolic pathways, and performance of nanomaterials in complex physiological environments comprehensively. Additionally, to achieve clinical translation of this technology, optimization of the nano-drug delivery system is essential not only for enhancing targeting ability and stability but also for developing cost-effective solutions that are convenient and suitable for low-resource areas.

**FIGURE 3:**
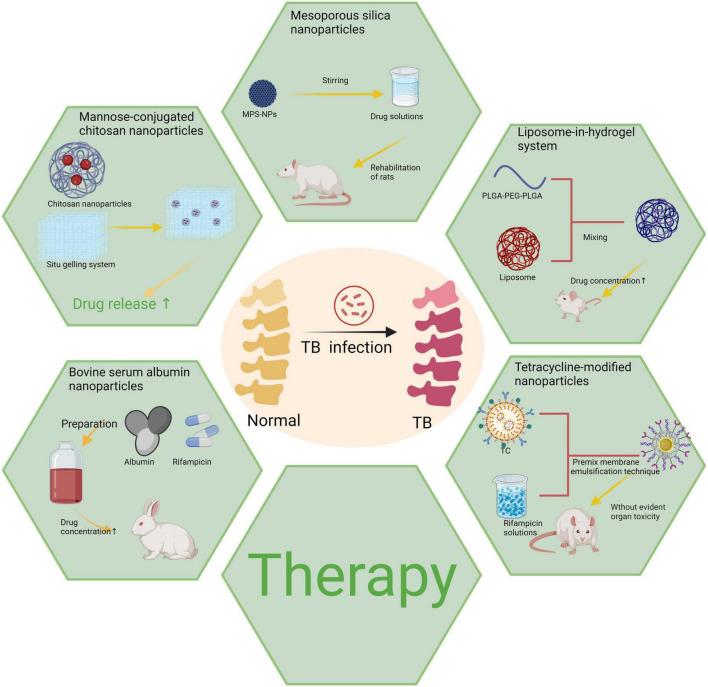
Various types of nanoparticles utilized in the treatment of bone and joint tuberculosis. Created in BioRender. ding, y. (2025) https://BioRender.com/n85l599.

With its versatility and interdisciplinary characteristics, nanotechnology has opened up a new avenue for precise diagnosis and treatment of bone and joint tuberculosis. From chitosan and bovine serum albumin to mesoporous silica and liposome hydrogels, various nanomaterials have demonstrated significant clinical potential through continuous optimization. These materials not only exhibit notable advantages in drug delivery systems, such as targeted delivery, prolonged drug retention time, and reduced toxic side effects but also facilitate bone tissue regeneration via nanoscaffolds, thereby providing a robust complement to address the limitations of conventional treatment methods (Figure 3).

The development direction of nanomaterials in the diagnosis and treatment of bone and joint tuberculosis is a topic of current interest. Research has demonstrated that nanomaterials offer cutting-edge solutions to overcome the limitations associated with traditional therapies, such as enhancing drug targeting, prolonging drug release time, improving drug efficacy, and enhancing biocompatibility. Notably, precise delivery of chitosan nanoparticles, targeted effect of bovine serum albumin nanoparticles, and synergistic sustained release system involving mesoporous silica and liposome-hydrogel have exhibited significant application advantages.

However, the treatment of bone and joint tuberculosis remains complex and challenging as an infectious disease. Future research should focus on deepening basic scientific exploration, optimizing nanomaterial design, and expediting clinical trial promotion. Additionally, interdisciplinary collaboration will be crucial for driving technological innovation in this field by integrating research findings from various disciplines. By leveraging nanotechnology’s potential to provide comprehensive and accurate solutions in the diagnosis and treatment of bone and joint tuberculosis, more patients can benefit while advancing global medical technology.
